# Unveiling the Role of Selenium in Child Development: Impacts on Growth, Neurodevelopment and Immunity

**DOI:** 10.3390/jcm14041274

**Published:** 2025-02-14

**Authors:** Gulnara Batyrova, Gulaim Taskozhina, Gulmira Umarova, Yeskendir Umarov, Marina Morenko, Bakhtiyar Iriskulov, Khatimya Kudabayeva, Yerlan Bazargaliyev

**Affiliations:** 1 Department of Clinical Laboratory Diagnostics, West Kazakhstan Marat Ospanov Medical University, Aktobe 030019, Kazakhstan; batyrovagulnara77@gmail.com; 2 Department of Evidence-Based Medicine and Scientific Management, West Kazakhstan Marat Ospanov Medical University, Aktobe 030019, Kazakhstan; 3 Department of Natural Sciences, West Kazakhstan Marat Ospanov Medical University, Aktobe 030019, Kazakhstan; eskendir.um@gmail.com; 4 Department of Children’s Diseases, Astana Medical University, Astana 010000, Kazakhstan; morenko.m@amu.kz; 5 Department of Normal and Pathological Physiology, Tashkent Medical Academy, Tashkent 100109, Uzbekistan; biriskulov@yahoo.com; 6 Department of Internal Diseases 1, West Kazakhstan Marat Ospanov Medical University, Aktobe 030019, Kazakhstan; h.kudabaeva@zkmu.kz (K.K.); y.bazargaliyev@zkmu.kz (Y.B.)

**Keywords:** selenium, selenoproteins, child development and growth, children, neurodevelopment, thyroid function, immune regulation, bone metabolism, Se deficiency, Se toxicity, antioxidant defense

## Abstract

Selenium (Se) is a vital trace element for children, playing a crucial role in numerous physiological processes, including antioxidant defense, immune regulation, thyroid function, and bone metabolism. Emerging evidence highlights its potential impact on child development and growth while also underscoring the complexity of its mechanisms and the global variations in Se intake. The aim of this review is to comprehensively elucidate the significance of Se in various biological processes within the human body, with a focus on its role in child development and growth; its biochemical effects on the nervous system, thyroid function, immune system, and bone tissue; and the implications of Se deficiency and toxicity. This review integrates findings from experimental models, epidemiological studies, and clinical trials to explore Se’s role in neurodevelopment, growth regulation, and immune competence in children. Selenoproteins, which regulate oxidative stress and thyroid hormone and bone metabolism, are essential for normal growth and cognitive development in children. Se deficiency and toxicity has been linked to impaired immune function, growth retardation, and decreased immune function. The findings underscore Se’s influence on various biological pathways that are critical for healthy child development and its broader importance for child health. Public health strategies aimed at optimizing selenium intake may play a pivotal role in improving pediatric health outcomes worldwide.

## 1. Introduction

Selenium (Se) is an essential trace element with significant biological roles in maintaining human health [1]. Se is a trace element that occurs naturally in the environment [2]. The environment, especially the atmosphere, is continually enriched with Se, and approximately 37–40% of the total atmospheric Se emissions are attributed to anthropogenic activities [3].

Se is unevenly distributed in soils worldwide, and many soils have low levels. The average Se content in soils worldwide is 0.4 mg/kg [4]. Soils in many countries contain Se, particularly in the Great Plains of the United States, Canada, South America, Australia, India, China, and Russia [5]. Brazilian soils have also been shown to be deficient in Se [6], as Se concentrations range from 10 to 150 μg kg^−1^ in Brazilian soils [7].

Se is found in small amounts in water in the form of selenates and selenites [8]. The Se content in groundwater is much higher than in seawater, which is mainly due to leaching of selenium from parent rocks and excessive fertilization of the soil with additives rich in selenium compounds [9]. Shallow groundwater has high Se concentrations (10–40 μg/L) because Se is mobilized from the soil and transported to shallow aquifers due to the prevailing oxidizing conditions there. People obtain selenium from drinking water [10]. According to the World Health Organization (WHO, 2011), the maximum permissible concentration of Se in drinking water is 50 μg/L [11]; in contrast, the European Union (EU) recommends a more stringent limit of 10 μg/L [12].

Dietary Se deficiency in humans results in growth retardation, impaired bone metabolism, thyroid dysfunction, and decreased immunity. Se deficiency occurs in some regions of the world. The prevalence of Se deficiency exceeds 60% in eight sub-Saharan African countries. Se deficiency is common among children and women in sub-Saharan Africa and is a possible contributor to stunting in children and reduced fertility in women. It is estimated that one in seven people worldwide have low dietary Se intake [13]. Moreover, the prevalence of Se deficiency is estimated to be 28% [14]. Se deficiency varies among populations depending on geological, geochemical, and climatic factors [15]. In Malawi, high levels of Se deficiency were found: its prevalence was 62.5% among women of reproductive age and 52.5% among adult men with plasma Se concentrations below the deficiency threshold. In parts of Ethiopia [16], particularly the Amhara region, in a study of school-aged children (n = 555), 49% of children also reported Se deficiency [17]. According to current data from the World Health Organization, one of the countries with the highest prevalence of Se deficiency is China [18], where approximately 72% of the country’s total land area is classified as Se-deficient, leading to Se deficiency in over 70 million individuals [19]. Thus, Se deficiency is widespread, and approximately 0.5–1 billion people worldwide suffer from Se deficiency [20]. The beneficial intake of Se for human health is approximately 50–400 μg/day [21]. According to the World Health Organization (WHO), the recommended daily intake for adults is 55 μg of Se [22]. For pregnant and lactating women, the requirement increases to 60–70 μg/day, depending on age. For children, the recommended intake ranges from 15 to 40 μg/day. The tolerable upper intake level (UL) for Se in children and adolescents varies by age and average body weight. It is set at 70 μg/day for children aged 1–3 years, 95 μg/day for ages 4–6 years, 130 μg/day for ages 7–10 years, 180 μg/day for ages 11–14 years, and 230 μg/day for adolescents [23].

The aim of this review is to comprehensively elucidate the significance of Se in various biological processes within the human body, with a focus on its role in children’s development and growth; its biochemical effects on the nervous system, thyroid function, the immune system, and bone tissue; and the implications of Se deficiency and toxicity.

## 2. Methodology

This review aims to investigate the impact of Se on child development and growth. A systematic approach to the literature search and analysis was used to achieve the stated objective. The literature search was conducted using the keywords “selenium” and “child development and growth” and “children” and “neuroprotective” and “selenium and thyroid function” and “immune system” and “selenium and bone metabolism” in PubMed, Scopus, and Web of Science databases. Inclusion criteria were original studies published in English that examined the effects of Se on children’s development and growth, cognitive function, thyroid function, immune function, and bone metabolism. Non-peer-reviewed works, as well as articles that were not relevant to the research topic, were excluded. The data were synthesized using a qualitative approach, focusing on key mechanisms and current research directions.

## 3. Bioavailability and Metabolism of Selenium

### 3.1. Selenium Absorption and Metabolic Incorporation

Se is one of the most abundant elements in the Earth’s crust, with an average concentration of 0.09 mg/kg [24]. It is an essential trace element for both humans and animals [25]. Se naturally occurs in two chemical forms: organic and inorganic [26]. Organic forms include selenomethionine (SeMet), selenocysteine (SeCys), and their methylated derivatives, while inorganic forms comprise Se salts such as selenate (SeO_4_^2−^) and selenite (SeO_3_^2−^) [27]. Selenomethionine is predominantly found in plant-based foods, including grains, legumes, and leafy green vegetables [28], whereas selenocysteine is primarily found in animal-based foods [29]. Major dietary sources of Se include cereals, nuts, meat, fish, seafood, dairy products, and Brazil nuts [30]. The exceptionally high Se content in Brazil nuts results from the significant presence of sulfur-containing amino acids in their proteins, allowing selenomethionine to non-specifically substitute methionine [31]. Inorganic Se, such as selenite and selenate, is commonly used in dietary supplements or naturally occurs in plants and fish [32]. Within the cell, selenate, an oxidation product derived from food, undergoes reduction to selenite and subsequently to hydrogen selenide through a series of redox reactions mediated by thioredoxin reductase or reduced glutathione (GSH) [33]. While hydrogen selenide plays a critical role, its inherent toxicity at excessively high concentrations poses a potential risk [34].

Dietary selenium is highly beneficial, with approximately 80% of Se consumed through food being absorbed, depending on the type of food and its chemical form [35]. Organic Se forms are absorbed more efficiently, typically via passive diffusion along a concentration gradient without specialized regulatory mechanisms [36]. Selenomethionine, absorbed in the intestines through methionine transporters, integrates into the body’s methionine pool and accumulates in tissue proteins in higher quantities than selenocysteine or inorganic forms [37]. It is metabolized similarly to methionine, with Se substituting sulfur and undergoing conversion to selenide through transamination and decarboxylation [38]. Se in soils originates from the erosion of selenide- and selenite-containing rocks and their binding to sulfide minerals [39]. It exists in soils as Se salts, iron selenite, elemental Se, or organic Se forms [40]. Soil Se levels are critical for predicting selenium availability in food and the resulting dietary Se intake in human populations [41]. Fluctuations in soil Se concentrations directly influence dietary Se levels, potentially resulting in either Se deficiency or toxicity [42]. Trace element metabolism is complex and dynamic, with the bioavailability of each element playing a critical role. The absorption of Se and other micronutrients is determined by multiple factors, including dietary intake, chemical form, gastrointestinal solubility, physiological status, and overall bioavailability [43].

### 3.2. Selenium Homeostasis and Regulation of Metabolism

The absorption of dietary Se occurs primarily in the gastrointestinal tract, specifically in the small intestine, from where it is transported to the liver for selenoprotein synthesis [44]. The dietary Se absorbed by the body is transported from enterocytes into the portal circulation and contributes to the biosynthesis of intestinal selenoproteins [44]. Subsequently, Se is distributed to various tissues throughout the body [45]. Notably, in the small intestinal enterocytes, selenomethionine (SeMet) is absorbed through an active mechanism involving the neutral amino acid Na^+^ transport system [46]. Selenoamino acids serve as substrates in the enzymatic intracellular metabolic pathway responsible for the production of endogenous hydrogen selenide. Selenocysteine is synthesized from selenomethionine via transsulfuration and subsequently converted into hydrogen selenide by the action of selenocysteine β-lyase (SCLY) [34]. SCLY provides selenide, a critical component required for selenoprotein biosynthesis within Se metabolism [47].

Selenate is proposed to be taken up by anion exchangers of the SLC26 family through passive transport mechanisms [48]. The solute carrier family 26 (SLC26) constitutes a functionally diverse group of anion transporters. Members of the SLC26 family facilitate the transport of various anions, including chloride, bicarbonate, and small organic dicarboxylates such as sulfate, fumarate, and oxalate [49].

All Se species absorbed by enterocytes are metabolized into hydrogen selenide (H2Se), which is transported. Subsequently, H2Se is converted into selenophosphate (SePhp), which serves as a precursor for the synthesis of selenoprotein [50]. Selenoproteins play a critical role in various biological functions of Se, with nearly all of them identified as redox enzymes [51]. These enzymes play a crucial role in redox homeostasis and Se metabolism, owing to their ability to interact with low-molecular-weight metabolites [52]. Among these are glutathione peroxidase (Gpx) enzymes, which are distributed across different intracellular and extracellular compartments, each exhibiting distinct substrate specificities [53]. Accordingly, these enzymes regulate hydrogen peroxide and other hydroperoxides, protecting them from oxidative damage as a result of their signaling effects [54]. All three selenoprotein iodothyronine deiodinases regulate the active form of thyroid hormones within tissues [55]. Additionally, two selenoproteins are directly involved in Se metabolism: selenophosphate synthetase-2, which converts Se into monoselenophosphate—a key intermediate in the synthesis of selenocysteine—and selenoprotein P (Sepp1), which facilitates the transport of Se from the liver to other tissues [56].

**Figure 1:**
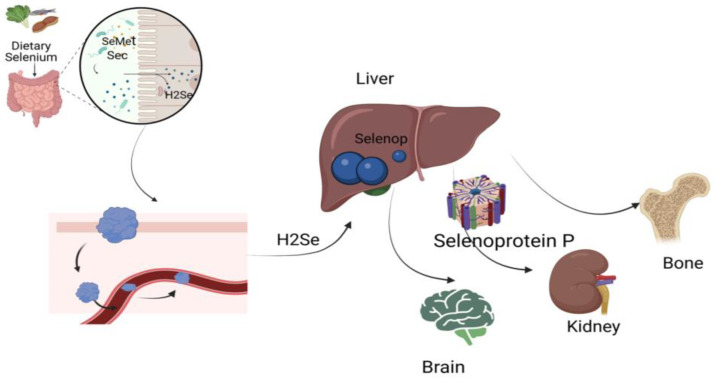
Selenium (Se) metabolism in the human body [61]. The metabolism of dietary Se, including its absorption, transport, and distribution. Se from food (e.g., selenomethionine and selenocysteine) is absorbed in the intestine, converted to hydrogen selenide (H_2_Se), and transported to the liver, where it is used for selenoprotein synthesis, including selenoprotein P. Selenoprotein P facilitates Se transport to target tissues such as the brain, kidneys, and bones, supporting essential biological functions.

After intestinal absorption, Se is rapidly taken up by the liver, where the transsulfuration pathway is more active than in other tissues [57]. This is because the liver serves as the primary organ through which Se, derived from selenomethionine, is incorporated into the selenium-specific pool [58]. Furthermore, the liver supplies other tissues with stored Se by secreting selenoprotein P (Sepp1) into the systemic circulation [59]. Selenium is transported primarily by selenoprotein P (SELENOP) via endocytosis involving apolipoprotein E receptor 2 (apoE2) and megalin to other tissues such as the brain, kidney, testes, and bone [60] (Figure 1). Se metabolism leads to its excretion through feces, breath, saliva, hair, and the urinary tract. The primary urinary metabolites include trimethylselenium ions, Se sugars, and Se-methylselenoneine [45].

## 4. The Significance of Selenium and Growth

**Figure 2:**
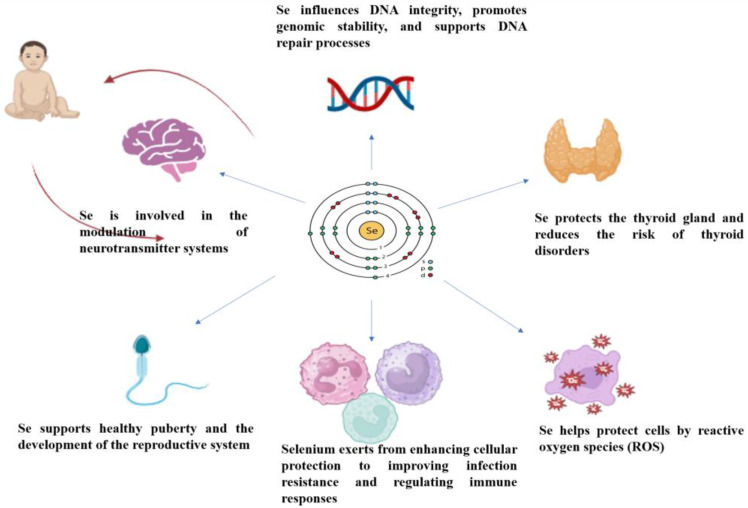
The role of selenium (Se) in child development and growth [74]. Se is involved in the regulation of neurotransmitter systems, which are critical for cognitive and neural development. Se protects the thyroid gland by contributing to the synthesis and regulation of thyroid hormones (T3 and T4), reducing the risk of thyroid disorders. Se supports healthy puberty and the development of the reproductive system. This is symbolized by a transition from childhood to adolescence, with representations of reproductive organs and hormonal signaling. Se plays a critical role in antioxidant defense by neutralizing ROS. Cells are depicted as shielded from oxidative damage, highlighting selenium’s contribution to cellular health. Selenium enhances immune responses, protecting against infections and modulating immune cell activity.

Se is an essential trace element that plays a critical role in development and growth and is important for overall health, supporting the function of the thyroid gland, immune system, and physiological homeostasis [27,62] (Figure 2). In humans, approximately 25 genes encode various selenoproteins, most of which are involved in redox processes [63]. A unifying characteristic of these proteins is their role in redox reactions; however, they exhibit significant diversity in tissue distribution and physiological pathways, indicating a broad range of substrate specificities and functions. Se plays a crucial biological role in living organisms primarily through its incorporation into a specific group of proteins known as selenoproteins [64]. The physiological functions of selenium are intrinsically linked to selenoproteins, which rely on the incorporation of selenocysteine (Sec) for their activity [65]. Selenocysteine, often referred to as the 21st amino acid [10], is a Se-containing analog of cysteine synthesized from tRNA-bound serine [66]. Selenocysteine transfer RNA (tRNA Ser/Sec) is a pivotal molecule in the biosynthesis of selenium-containing proteins, playing a crucial role in regulating their production [67]. Mutations substituting Sec with any other amino acid residue lead to enzyme inactivation, underscoring Sec’s essential role in various biological processes [68]. This suggests that Sec-containing proteins are largely responsible for the health benefits associated with Se [69]. Selenoproteins function by reducing oxidative stress caused by viral pathogens, with Se serving as a redox center in regulating key redox activity enzymes such as thioredoxin reductases (TrxR1, TrxR2, TrxR3), methionine-R-sulfoxide reductase 1 (MSRB1), and selenoproteins such as selenoprotein H (SELENOH), selenoprotein M (SELENOM), and selenoprotein W (SELENOW) [70]. Additionally, glutathione peroxidase (GPx) is a key enzymatic antioxidant that plays a critical role in mitigating oxidative stress. It detoxifies peroxides by converting them into non-toxic compounds, thereby contributing significantly to the cellular antioxidant defense system [71], and iodothyronine deiodinases (DIO1, DIO2, DIO3) play important roles in thyroid hormone metabolism [72]. At the cellular level, the selenoenzyme thioredoxin reductase maintains the intracellular redox state by participating in deoxyribonucleic acid (DNA) synthesis through nucleotide reduction [73]. These functions underscore the critical role of Se in the development of the fetus, infant, and child.

## 5. Biochemical Interactions of Selenoproteins

### 5.1. Selenium’s Role in Antioxidant Defense and Redox Regulation

Among the (semi)metal trace elements, Se holds particular significance due to its unique biological role. Se is incorporated into proteins through a specialized synthesis pathway, encoding the 21st proteinogenic amino acid, selenocysteine, via the UGA codon in a process known as translational decoding [75]. This genetically encoded mechanism allows Se to be integrated into proteins as a component of selenocysteine, playing a crucial role in redox signaling [76]. In redox signals, selenoproteins are involved in antioxidant defense and regulation of the redox state [77]. The regulation of the redox state is mediated by the cell’s antioxidant system [78]. This system includes high-molecular-weight selenoenzymes, such as glutathione reductase peroxidase (Grx), which functions through nicotinamide adenine dinucleotide phosphate (NADPH), glutathione reductase, the glutathione (GSH) system, and glutathione peroxidases (Grx) [79]. GPX plays a pivotal role in modulating signaling cascades within cells by counteracting hydroperoxide stimulation, which can otherwise promote inflammation through cytokines or lipid mediators, disrupt insulin signaling, and contribute to various forms of programmed cell death [80]. One such protein, glutathione peroxidase 3 (GPx3), is a selenium-containing enzyme that plays a crucial role in maintaining redox system balance [81]. Furthermore, GPx4, a selenoprotein present in brain tissue, plays a pivotal role in cellular antioxidant defense [82]. Its transcriptional activity and expression are influenced by cellular Se levels, which enhance its antioxidant capacity under various oxidative stress conditions. GPx4 is also one of the genes regulated by the redox-sensitive transcription factor (Nrf2) transcription pathway [83].

The GSH system, a thiol-dependent antioxidant system, plays a critical role in protecting cells against oxidative stress by efficiently neutralizing free radicals [84]. A key mechanism of antioxidant defense involves the reaction of GSH with prooxidants. Beyond its antioxidant function, GSH metabolism supports additional roles, including participation in cell signaling, protein interactions, and defense against oxidants [85,86]. The thiol group of GSH directly interacts with various ROS, such as hydroxyl radicals, hydroperoxides, alkyl radicals, and superoxide anions [87]. Cellular GSH is integral to numerous biological processes, including protein and DNA synthesis, cell proliferation, growth, apoptosis, and cancer progression [88]. Furthermore, it is essential in regulating immune system functions, such as T-cell growth and metabolic reprogramming, as well as in xenobiotic metabolism, amino acid transport, and redox-sensitive signaling pathways [89].

In redox signaling, members of the NADPH oxidase (NOX) family serve as key intracellular generators of ROS [90]. While ROS are produced as toxic byproducts of metabolism, they also function as critical biomolecules in cell signaling [91]. At the cellular level, the thioredoxin (Trx) system plays a central role in modulating ROS levels and is vital for maintaining redox regulation [92]. The Trx system supports redox homeostasis in the cytosol, nucleus, and mitochondria [93]. It exhibits antioxidant functions by reducing ribonucleotide reductase and methionine sulfoxide reductase, regulating the activity of several redox-sensitive transcription factors, and contributing to DNA and protein repair [94]. Furthermore, the Trx system plays a significant role in supporting the immune system [95].

Thioredoxin reductases (TrxRs) are the sole enzymes responsible for enabling the redox activity of thioredoxin (Trx), catalyzing the NADPH-dependent reduction of both endogenous and exogenous compounds [96]. Mammalian TrxRs belong to a family of selenium-containing pyridine nucleotide-disulfide oxidoreductases, characterized by a conserved redox-active catalytic site with the sequence -Cys-Val-Asn-Val-Gly-Cys-. This conserved structure is essential for their enzymatic mechanism [97]. In mammals, TrxRs exist in three isoforms: cytosolic TrxR (TrxR1) [98], mitochondrial TrxR (TrxR2) [99], and testis-specific thioredoxin-glutathione reductase (TrxR3). All three isoforms of TrxRs possess a secondary redox-active site with broad substrate specificity [100]. This site, located in the C-terminal region, contains a conserved and flexible -Cys-SeCys- motif, where SeCys represents selenocysteine [101]. The presence of the SeCys residue is critical for the reductase activity of TrxRs. TrxR1, in particular, plays a key role in selenium metabolism and is closely linked to the overall function of selenoproteins [102]. TrxR1 is instrumental in regulating cell phenotype and mediating responses to growth and signaling events [103].

### 5.2. Selenium and Brain Development: Neuroprotective Effects

**Figure 3:**
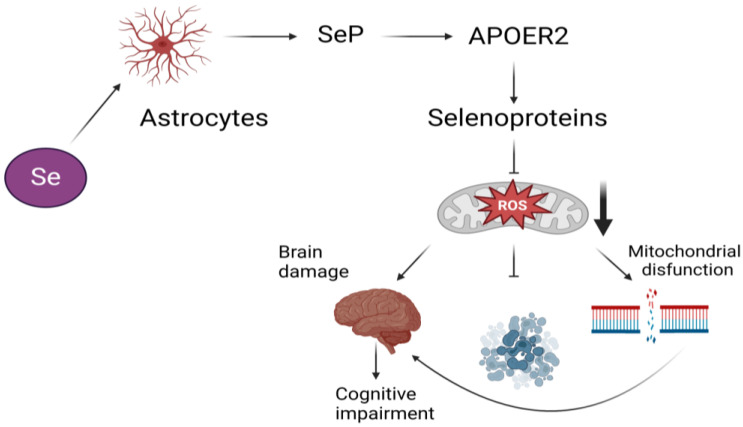
Selenium-associated pathways contributing to neural damage [104]. Selenium is transported to neurons via its incorporation into selenoprotein P (SELENOP), which interacts with its receptor, low-density lipoprotein receptor-related protein 8 (LRP8), also referred to as apolipoprotein receptor E2 (ApoER2). This mechanism plays a crucial role in maintaining antioxidant defenses by enabling selenoproteins to mitigate the harmful effects of reactive oxygen species (ROS). Dysfunction in selenium transport pathways can lead to the suppression of protective mechanisms, upregulation of harmful gene expression, and the onset of mitochondrial dysfunction resulting from DNA damage. Such impairments are closely linked to the development of neurological disorders, including cognitive impairment.

Se and selenoproteins play critical roles in brain signaling pathways [104] (Figure 3), with selenoprotein P (Sepp1 or SELENOP) serving as a selenium transporter containing 10 selenocysteine residues [105]. Plasma SELENOP facilitates Se transport from the liver to various tissues, including the brain and testes, where selenium is vital for normal brain function [106,107]. Several selenoproteins are widely distributed within the central nervous system, particularly in neurons [108].

Se delivery to neurons is mediated by SELENOP and its receptor, low-density lipoprotein receptor-related protein 8 (LRP8, also known as apolipoprotein receptor E2 [ApoER2]) [109]. SELENOP-bound selenium enters the brain via interactions with LRP8 at the blood–brain barrier (BBB) in brain capillary endothelial cells and in choroid plexus epithelial cells [107]. Within these pathways, SELENOP is absorbed in its oxidized state to ensure selenium availability for critical neurological functions [104]. SELENOP is crucial for the synthesis of selenoproteins in the brain, where it helps maintain selenium homeostasis by facilitating selenium transport and binding to other brain cells [110]. SELENOP expression has been implicated in neurodegenerative changes, particularly affecting various brain cell types, including neurons and ependymal cells, the latter being responsible for cerebrospinal fluid production [111].

The brain, as one of the most metabolically active organs, relies on selenium for protection against oxidative damage [112]. Oxidative stress is recognized as a critical contributor to selenium-related neurotoxicity, driving neuronal death and playing a central role in the pathogenesis of numerous neurological diseases [113]. Specifically, neurodegenerative and neuropsychiatric disorders are strongly associated with the interplay between oxidative stress and the antioxidant capacity of selenoproteins [114].

The expression of selenoproteins serves as a potent neuroprotective mechanism for neurons [115]. Neuroprotection can be defined as the maintenance of cellular and molecular interactions within the brain to prevent or mitigate neural dysfunction [116]. Se’s neuroprotective effects are mediated through two primary mechanisms. First, Se prevents the oxidation-induced activation of transcription factors [117]. Second, it directly reduces free radicals [118]. In the context of neuroprotection, selenoproteins play a critical role in mitigating oxidative stress by scavenging ROS and reactive nitrogen species (RNS) [119,120]. Furthermore, through a translation-dependent mechanism, selenium is incorporated into selenoproteins, where it regulates the balance between anti-apoptotic and pro-apoptotic signals. This regulation is achieved by influencing key kinases and transcription factors via specific protein–protein interactions [121].

Selenoprotein W (SelW) is a crucial member of the selenoprotein family, known for its protective role in safeguarding neurons from oxidative stress during neuronal development [122]. SELENOW, also referred to as selenoprotein W, is highly expressed in the brain, particularly in neurons, in both mice and humans, positioning it as one of the most abundant selenoproteins in the brain [123].

### 5.3. Selenium and Thyroid Function in Growth Regulation

Se is a critical trace element in the synthesis, activation, and metabolism of thyroid hormones, ranking second in importance only to iodine [124]. As a central component of thyroid hormone function [62], Se plays a vital role in supporting processes such as growth, development, differentiation, and metabolism throughout the body [125]. Se is a vital trace element involved in maintaining the homeostasis of various endocrine functions, particularly those related to thyroid signaling pathways [62]. These pathways, when activated by excessive ROS, may share common triggers that contribute to the pathogenesis of thyroid diseases at different stages [126]. In mitochondria, numerous oxidation processes occur continuously, especially in organelles where Se-dependent enzymes serve as cofactors. Mitochondria not only facilitate ATP production within cells but also represent the primary source of ROS generation [127]. Reactive oxygen species (ROS) and excess hydrogen peroxide (H_2_O_2_), produced by thyroid follicles during thyroid hormone biosynthesis, are mitigated by cellular antioxidant defense systems [128]. These systems include the glutathione peroxidase (GPx) and thioredoxin reductase (TxnRd) families, thyroid hormone deiodinases, and other selenoproteins involved in redox regulation [129]. Among these, glutathione peroxidase (GPx) plays a pivotal role in protecting thyroid cells from oxidative damage [80]. Of the eight glutathione peroxidases, five (GPx1–4 and GPx6) are selenocysteine-containing proteins [130].

Glutathione peroxidase 3 (GPX3), an antioxidant enzyme, plays a crucial role in mitigating oxidative stress within the thyroid gland. The GPX3 gene, located on chromosome 5 at the 5q32 region, spans approximately 10 kb and consists of five exons. It encodes a 23 kDa protein that forms a homotetramer [131] and exhibits enzymatic kinetics similar to GPx1, specifically regarding H_2_O_2_ metabolism. In human thyrocytes, GPx3, which is primarily extracellular, represents the most abundant selenoprotein and acts as a direct regulator of thyroid hormone synthesis [132]. In the absence of thyroid-stimulating hormone (TSH) at the apical pole, GPx3 secretion reduces H_2_O_2_ levels required for iodination reactions, thereby moderating oxidative stress during hormone synthesis [133].

The thioredoxin (Trx) molecule functions as an electron donor, stabilizing unpaired electrons from free radicals [134]. The oxidized form of Trx is regenerated by the action of thioredoxin reductase (TrxR), using NADPH as an electron source, which is converted to its reduced form in the process [135]. Additionally, TrxR acts as a growth factor, exerting various biological effects either directly on the cell or through interactions with thioredoxin. The activation of signaling pathways in thyrocytes is linked to increased H_2_O_2_ production, which, in turn, stimulates the synthesis of thioredoxin reductase [136].

Selenoprotein-containing homodimeric thioredoxin folds regulate thyroid hormone signaling in a time- and cell-specific manner through the action of deiodinases. These enzymes mediate both the activation and inactivation of thyroid hormones. There are three types of deiodinases—type 1 (DIO1), type 2 (DIO2), and type 3 (DIO3)—each differing in catalytic properties and tissue distribution [137]. Notably, tissue-specific deiodinases allow precise regulation of thyroid hormone signaling within individual cells without altering systemic thyroid hormone concentrations [138]. The induction of DIO1 activity by T3 occurs via a classical DR4+2 thyroid hormone response element (TRE) in the DIO1 promoter [139]. DIO2 expression is strongly induced by thyroid-stimulating hormone (TSH) receptor activation via the cyclic AMP (cAMP)-responsive element (CRE), which stimulates adenylate cyclase-protein kinase A (PKA) signaling pathways [140]. DIO2 also converts the prohormone T4 to the biologically active hormone T3, thereby activating thyroid hormones [141].

In contrast, DIO3 inactivates both T4 and T3, thereby halting thyroid hormone activity [142]. Collectively, deiodinases regulate intracellular T3 levels and gene expression on a cell-specific basis, playing critical roles in maintaining thyroid hormone homeostasis and mediating processes such as development, growth, and metabolism [143].

### 5.4. Selenium and Immune System Development

**Figure 4:**
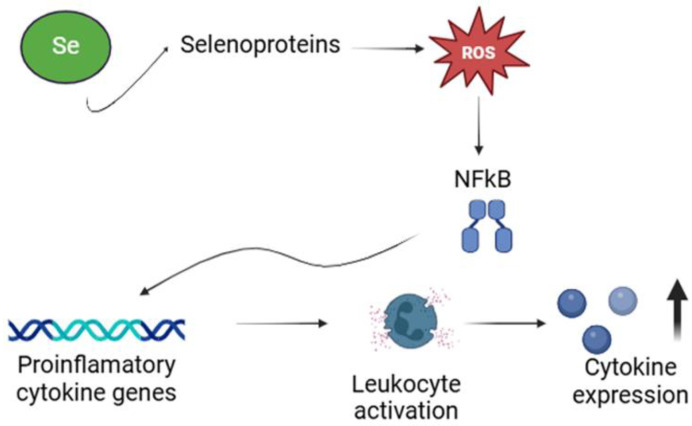
The role of selenium (Se) in metabolic pathways of inflammation [166]. The figure illustrates the role of selenium in mitigating oxidative stress at the cellular level through its incorporation into selenoproteins. Se modulates the NFkB signaling pathway, a key regulator of pro-inflammatory cytokine genes. This activation of leukocytes leads to increased cytokine expression.

Se plays a vital role in the immune system, primarily due to its redox, anti-inflammatory, and antioxidant properties [144]. Selenocysteine, a key component of selenoproteins, significantly reduces inflammation and oxidative stress [145]. Oxidative stress arises from an imbalance between the production of ROS and the body’s antioxidant defenses [146]. ROS are a critical driver of inflammatory responses, as the activation of redox pathways enhances the expression of transcription factors and inflammatory cytokines [147]. Se’s anti-inflammatory effects are mediated through its inhibition of nuclear factor kappa-light-chain-enhancer of activated B cells (NF-κB), a key regulator of pro-inflammatory responses, thereby helping to maintain homeostasis in infected tissues [148]. ROS play a crucial role in immune defense, particularly in microbial killing by phagocytes [149]. During phagocytosis, ROS generated by macrophages and neutrophils are essential for the oxidative destruction of pathogens [150,151]. Moreover, ROS serve as critical mediators of cell signaling and intercellular communication in both phagocytic and non-phagocytic immune cells [152]. Se also increases the activity of glutathione peroxidase (GPx) in lymphocytes [153]. Through its antioxidant properties, GPx attenuates oxidative stress, thereby modulating inflammatory processes and supporting immune system functionality [154]. Se levels significantly influence the balance between cellular and humoral immunity and between type 1 and type 2 helper T cells [155]. Maintaining adequate Se concentrations is essential for maintaining normal cellular functions and overall immune health in humans [156]. Reduced levels of selenoproteins in tissues and cells can lead to disruption of normal physiological functions and, in some cases, cell death [69]. Thus, the effects of Se on key immune functions occur through various interactions with ROS, nuclear transcription factor κB (NF-κB), ferroptosis, and nuclear factor erythroid 2-related factor 2 (NRF2) signaling pathways [157]. By modulating the NRF2-HO1 pathway, selenium helps to inhibit oxidative stress and enhances the expression of downstream antioxidant enzymes such as GPx and superoxide dismutase (SOD) [158]. NRF2 plays a crucial role in the oxidative stress response, protecting cells from oxidative damage [159]. NF-κB activation occurs through two primary pathways: canonical and non-canonical [160,161]. The canonical pathway is initiated in response to pro-inflammatory cytokines and involves the phosphorylation of the IκB kinase (IKK) complex [162]. This phosphorylation triggers the degradation of IκB proteins, allowing NF-κB to translocate to the nucleus and activate the transcription of target genes [163]. In contrast, the non-canonical pathway does not rely on IκBα degradation but depends on the processing of the precursor protein p100 (NF-κB2) and is activated by specific stimuli [160]. Canonical NF-κB plays a central role in all aspects of immune responses [164]. Notably, there is evidence that the canonical and non-canonical NF-κB pathways work in concert, forming an additional signaling axis to regulate specific functions within the adaptive immune system [165] (Figure 4).

### 5.5. Se and Bone Metabolism

Se is an essential trace element that plays a critical role in regulating and maintaining bone metabolism [167]. Bone metabolism is regulated through a dynamic and continuous interplay between bone resorption and formation [168]. Bone formation, mediated by osteoblasts, and bone resorption, mediated by osteoclasts, constitute a tightly regulated and balanced process that ensures the constant renewal of skeletal tissue [169]. Se exerts a protective effect on the skeletal system and supports bone tissue metabolism [170]. Se enables the regulation of bone metabolism through its incorporation into selenoproteins [171]. Selenoproteins mediate the physiological functions of Se, including its antioxidant activity and the maintenance of redox balance in regulatory processes that govern bone cell proliferation and differentiation [172]. Bone homeostasis is regulated by the coordinated activity of osteoblasts and osteoclasts [173]. The balance between bone formation and resorption is controlled by key regulatory pathways, among which the OPG/RANK/RANKL system plays a pivotal role in bone metabolism [174]. Within this system, osteoprotegerin (OPG), a member of the tumor necrosis factor (TNF) receptor family, has been identified as a critical component secreted by osteoblasts. OPG protects the skeleton from excessive bone resorption by functioning as a soluble decoy receptor that binds to RANKL. This binding prevents RANKL from interacting with the RANK receptor. Elevated expression of the gene encoding OPG has been shown to result in increased bone mass and a reduction in both the number and activity of osteoclasts [175].

In most cases, the RANKL/RANK/OPG signaling pathway is influenced by the modulation of ROS. ROS are continuously generated in small quantities by healthy cells and are effectively managed by various antioxidant systems. Oxidative stress arises when the delicate balance between ROS production and elimination is disrupted [176]. Oxidative stress adversely affects normal bone physiology by increasing ROS levels in the skeletal system. Excess ROS inhibit osteoblast differentiation and promote osteoclastogenesis, partly through NFκB-independent pathways that stimulate osteoclast differentiation. Additionally, ROS enhance RANKL expression in osteoblasts, facilitating crosstalk between osteoblastic and osteoclastic lineages and further driving osteoclastogenesis [177]. Since oxidative stress has a strong impact on osteoblast activity and mineralization, an imbalance between ROS production and antioxidant defenses adversely affects bone metabolism [178]. Se supports bone health by safeguarding bone metabolism through its antioxidant mechanisms, thereby contributing to growth.

## 6. Toxic Effects of Selenium

Se is an essential trace element required for normal human growth and development. However, similar to other trace elements, it can exhibit toxic effects at elevated concentrations [179]. The impact of high Se concentrations on human health is significant in South and North America, India, China, and Italy. The high Se content in these regions, mainly due to their geological origin, also indicates unusually high Se levels in local foods and sometimes in ambient air and drinking water, which ultimately affects human health [180]. To ensure human safety, the tolerable maximum total intake level (TIL) of Se is set at 400 μg/day [181]. The no observed adverse effect level (NOEAL) of dietary Se is 1540–1600 μg/day [182]. Based on the Se and Vitamin E Cancer Prevention Study (SELECT), the lowest observed adverse effect level (LOAEL) for Se in humans was found to be 330 μg/day, and such intakes are associated with an increased risk of alopecia, an early sign of Se toxicity [183]. Accordingly, the dose-dependent biotoxicological response of Se depends mainly on the chemical type and metabolic activity of each Se compound [10]. The toxic effects of Se are observed at levels slightly exceeding its homeostatic requirements [184], and it exerts cytotoxic (cell cycle arrest and cell growth inhibition) and genotoxic (DNA damage) effects [185]. Its toxicity is also known to be related to its chemical similarity to sulfur, its ability to displace protein during assembly, and oxidative stress [186].

Se genotoxicity is manifested by the interaction of excess ROS with cellular components that are genotoxic to cells [187]. Base damage occurs through reactions between deoxyribose sugars in the deoxyribose chain of nucleic acid (DNA) and the nitrogenous bases of DNA, which also leads to its breakage [188]. In addition, ROS cause DNA damage. As a result, cells with the most DNA damage die by necrosis or apoptosis [189]. The most important source may be oxidized DNA produced by dying cells. However, Se is believed to threaten the stability of genetic information by interfering with DNA repair and transcriptional regulation [190]. Other molecular mechanisms associated with Se genotoxicity include interactions with tissue thiols such as glutathione [191]. As a result, Se oxidizes thiols, increasing the likelihood of ROS formation and oxidative damage to the body [80].

Se also has cytotoxic properties that cause irreversible changes in cells, as evidenced by the ability of Se to increase the production of ROS in cells [192]. Excessive accumulation of ROS damages mitochondrial membrane potential, lipids, and proteins [127]. In addition, ROS-induced oxidative stress develops as a result of activation of the mitochondrial apoptotic pathway [193]. ROS not only act as modulators of signaling pathways influencing various biological processes but also exert cytotoxic effects involving c-Jun N-terminal kinases (JNKs), a subgroup of mitogen-activated protein kinases [194]. JNKs regulate cell proliferation, differentiation, apoptosis, and numerous other cellular functions through activation [195]. ROS can stimulate JNK via tumor necrosis factor and modulate signaling pathways affecting critical processes such as cell growth, apoptosis, and cell adhesion [196]. Se is closely related to the redox potential that causes cytotoxic effects. Se may also exert cytotoxic effects due to its ability to modulate cellular signaling pathways through the thiol reduction system, particularly by generating ROS and affecting the expression of correlated genes and proteins.

## 7. Animal Studies on Selenium Deficiency and Toxicity

Several studies have been conducted on the effects of Se deficiency in animal models. A study evaluating the role of Se in supporting brain development, behavioral plasticity, and maturation examined the effects of different isocaloric diets—optimal, suboptimal, and Se-deficient—administered to pregnant and lactating female rats and their offspring for 40 days postpartum. The findings revealed early adverse behavioral changes in juvenile rats from the suboptimal group, suggesting that even moderate Se deficiency during early life may lead to sex-specific impairments in optimal brain development [197]. A study investigating the chronic administration of sodium selenate, a non-toxic selenium compound, reported significant changes in behavioral phenotypes in tau mouse models [198]. In addition, the study demonstrated that a Se-deficient diet elevated the expression of inflammatory cytokines via activation of the iNOS/NF-κB pathway, resulting in inflammatory lesions in the pig brain, while HSPs were implicated in the compensatory regulation of the inflammatory response [199].

This study, which identifies a congenital deficiency of selenoprotein biosynthesis, highlights the important role of selenoenzyme deiodinase 2 (DIO2) in thyroid hormone metabolism, in particular its association with normal thyroid function. These results suggest an interaction between thyroid hormones and puberty [200]. Moreover, this study primarily investigated the effects of Se deficiency on thyroid hormone metabolism in mammals. The findings demonstrated that Se deficiency, in addition to inhibiting the conversion of thyroxine (T4) to triiodothyronine (T3), significantly reduced the expression levels of key thyroid hormone-metabolizing enzymes, including Dio1, Dio2, and Dio3, in chickens [201].

The results of the study showed that selenium deficiency significantly reduced selenoprotein gene expression and altered cytokine levels in the spleen of chickens, indicating its important role in immune regulation [202]. In addition, the study revealed that Se deficiency suppressed selenoprotein expression, leading to reduced levels of CD11c, CD40, CD86, and MHC II, thereby impairing the differentiation and immune function of chicken dendritic cells [203]. Furthermore, this study demonstrated that dietary Se deficiency not only reduces the expression of selenoprotein T (SelT) and its associated synthetases but also decreases antioxidant activity and impairs immune responses in the immune organs of broilers [204]. In a study on the effect of Se deficiency on peritoneal macrophage function in mice, it was observed that inflammatory factors, oxidative stress levels, inhibition of phagocytosis, and decreased phagocytic capacity were observed in primary cultured Se-deficient peritoneal macrophages [205]. Additionally, studies revealed that Se deficiency in chicken thymus tissue induced immunological alterations and immune stress, alongside changes in selenoprotein expression, which could contribute to oxidative stress [206]. Furthermore, a study demonstrated that low Se levels adversely affected intestinal mucosal immunity in commercial broilers, leading to reduced levels of duodenal mucosal soluble IgA and anti-inflammatory cytokines, including TGF-β1 and IL-10 [207].

Findings from this study indicate that Se deficiency-induced growth retardation is linked to impaired bone metabolism and the development of osteopenia in second-generation rats [208]. Furthermore, another study demonstrated that Se deficiency can result in reduced antioxidant activity, decreased epiphyseal plate thickness, and pathological changes in rats subjected to a low-selenium diet over two generations [209].

Although Se is an essential micronutrient, excessive intake can lead to a range of health problems due to Se toxicity [210]. Both animals and humans are susceptible to toxicity caused by excessive dietary Se [211]. Se toxicity is primarily linked to excessive daily intake from food and water sources. In addition, adequate (0.05–0.10 mg/kg) and toxic (4–5 mg/kg) doses of Se have been determined in the animal diet [212]. Based on animal studies, the NOAEL (no observed adverse effect level) values for Se with respect to body weight effects were determined to range between 0.24 and 1.2 mg Se/kg body weight/day. The toxic dose of Se in rats has been determined to be ≥1.2 mg Se/kg body weight per day, leading to increased mortality and reduced weight gain. Growth retardation was also observed following selenite supplementation at the NOAEL of 0.6 mg Se/kg body weight per day and Se-enriched wheat supplementation at 0.8 mg Se/kg body weight per day, as well as at higher doses [213].

Se toxicity in the environment is not solely determined by physical and chemical processes; rather, its toxic potential is shaped by the mobility, bioavailability, and accumulation of selenium compounds within the food chain [214]. In general, living organisms can absorb Se either through water or dietary intake. While humans and terrestrial animals primarily obtain Se through food, aquatic organisms, such as fish, assimilate soluble selenium directly from water. The World Health Organization recommends that drinking water contain less than 40 μg/L of total Se, primarily in the form of selenate (Se(VI)) and selenite (Se(IV)) oxo-anions [215]. However, both selenite and selenate are recognized as toxic [216]. While their removal from water is necessary, selenate (Se(VI)) is structurally more stable than selenite (Se(IV)), exhibiting lower reactivity and weaker adsorption by metal oxides. As a result, selenate is the predominant Se species in groundwater. Se compounds present in groundwater can bioaccumulate in sensitive aquatic organisms, potentially leading to toxic effects [215]. In aquatic fish, Se exposure leads to significant accumulation of Se in the gills. Fish are particularly vulnerable to consuming naturally occurring food organisms that bioaccumulate Se [179]. Se becomes highly toxic to fish only when its concentration exceeds the physiological threshold. The optimal dietary range for Se is 0.1–3 mg/g dry weight (dw), beyond which it exhibits toxic effects [217]. In addition, Se toxicity in white sturgeon diets occurs at concentrations above 20.5 mg Se/kg and is expressed as selenomethionine (Se-Met) [218].

A study investigating chronic Se exposure demonstrated that 90 days of exposure to Se-Met (at control levels of 3.6, 12.8, and 34.1 μg Se/g dry weight) significantly impaired behavioral responses in adult zebrafish. This prolonged exposure was associated with deficits in social learning, which were linked to dysfunction in the serotonergic system of the brain [219]. This study explored the neurotoxic effects of chronic Se intake on cognitive performance in adult zebrafish. Over a 30-day period, zebrafish were fed diets containing varying concentrations of L-selenomethionine: 2.3 (control), 9.7, 32.5, and 57.7 μg Se/g dry weight. The findings revealed that high dietary selenium doses (32.5 and 57.7 μg Se/g) significantly impaired cognitive performance [220].

This study indicates that Se toxicity impairs immune function by promoting inflammation through activation of the NF-κB pathway and disrupting the Th1/Th2 balance in the chicken spleen [221]. In addition, excessive Se supplementation in chickens has been shown to decrease immunity, increase oxidative damage, and cause pathological changes, including thymic reticular cell degeneration, particularly at high dietary levels and prolonged exposure [222].

In this study, adult rats were exposed to 5 mg Na_2_SeO_3_/L in drinking water for 90 days. The findings demonstrated that selenium exposure significantly reduced bone mass, femur length, and cortical bone thickness, while also negatively affecting the macroscopic and microscopic structures of femoral tissue in adult male rats [223].

## 8. Animal Studies Assessing Se Supplementation Impacts on Health

Experimental studies have demonstrated that Se is a critical trace element involved in numerous biological processes. Notably, the diverse effects of Se supplementation have been extensively investigated across various experimental models.

Study results demonstrated that Se supplementation in chicken diets reduced the shedding of the low-pathogenicity avian influenza virus (H9N2), enhanced innate antiviral immune responses, and increased the tissue expression of interferons and interferon-stimulated genes [224]. This study demonstrates that dietary Se enhances selenoprotein synthesis, particularly by optimizing stress-responsive selenoproteins and modulating immune function through the regulation of IFN-γ and IL-6 expression [225]. This study demonstrated that dietary Se at adequate levels supports the body’s antioxidant system. Furthermore, pigs supplemented with organic Se showed reduced susceptibility of muscle fibers to oxidative stress compared to other groups [226].

Recently, other studies have shown that organic Se supplementation improves growth performance and slaughter characteristics in naked-neck chickens [227].

These studies have demonstrated that Se supplementation enhances antioxidant levels in chicks, thereby supporting their growth potential [228]. Another study reported that dietary supplementation with 0.45 mg/kg selenium yeast enhanced eggshell strength in aged laying hens [229]. Based on this study, dietary supplementation with 0.133 mg Se/kg of selenium yeast optimized broiler growth performance. Additionally, while sodium selenite (SS) enhanced antioxidant enzyme activity, selenium yeast (SY) demonstrated higher selenium bioavailability, particularly in feather and carcass retention, making it a more effective long-term selenium source for tissue mineralization [230] (Table 1).

## 9. Human Studies on Se Deficiency and Toxicity

Se, an essential trace element for child development, plays a pivotal role in various biological processes, including neuroprotection, thyroid hormone metabolism, immune function, and bone metabolism. Maintaining an adequate selenium status is crucial for children, as its deficiency can adversely impact skeletal development, growth, and overall health. Conversely, selenium toxicity is equally detrimental to health.

This study investigated the association between anthropometric characteristics, iron biomarkers, and serum Se levels with cognitive performance in preschool children in rural Ethiopia. Furthermore, cognitive impairment was significantly associated with growth retardation, anemia, and Se deficiency [231].

Moreover, studies showed that Se deficiency was associated with elevated serum thyroxine levels in more than half of children with severe iodine deficiency in the Amhara region of Ethiopia, suggesting potential implications for thyroid metabolism and iodine supplementation programs [17].

Additionally, this study also identified alterations in the gut microbiome of children with Kashin–Beck disease (KBD), a condition associated with Se deficiency, suggesting a potential interaction between gut microbiota composition, nutrient absorption, and environmental factors, including Se deficiency, in the pathogenesis of KBD [232].

This study suggests that Se deficiency in children remains a major factor in the development of Kashin–Beck disease, as evidenced by persistent dietary Se deficiency and mercury-induced disease progression despite previous efforts to restore Se status in endemic areas of China [233]. In this study, Se deficiency was linked to altered expression of enzymes involved in chondroitin sulfate (CS) sulfation in the distal articular surface of proximal interphalangeal joints from school-aged children with Kashin–Beck disease (KBD), leading to a significant reduction in the total number of chondrocytes across the superficial, middle, and deep cartilage layers [234].

In humans, Se toxicity is linked to excessive selenium levels in certain soils or the consumption of megadoses of Se supplements, resulting in selenosis characterized by symptoms such as hair loss, dermatitis, and irritation [10]. The Se content in foods varies significantly, necessitating careful monitoring to avoid Se toxicity. Major dietary sources of selenium include fish meal, seafood, dried brewer’s yeast, kidneys, liver, eggs, whole grains, and nuts [235]. Se is predominantly found in food sources such as yeast (500–4000 μg/g), fish (0.06–0.63 μg/g), and Brazil nuts (0.2–512 μg/g) [236]. Although Brazil nuts offer significant health benefits, their consumption should be moderated. A serving of 30 g (approximately six nuts) can exceed the recommended daily intake of Se (400 μg) and may even surpass the threshold for Se toxicity (1200 μg). To ensure safe Se consumption, reducing the portion size to 15 g (around three nuts) has been deemed safe [237]. Furthermore, to mitigate the risk of Se toxicity, it has been proposed that Brazil nuts originating from regions with high Se content should be clearly labeled on packaging. Additional measures, such as reducing package sizes and indicating the percentage of the recommended daily selenium intake, would also contribute to safer consumption practices [238]. Proof of this is shown in a study conducted among children living in the Brazilian Amazon, where 41 children aged 2 to 6.5 years from a public preschool participated as part of a public health program, receiving 15–30 g of Brazil nuts three times a week. A control group of 88 children who did not consume nut-enriched foods was also included. The average Se intake in children who consumed Brazil nuts was 155 μg/day, exceeding the tolerable upper intake levels (ULs) of 60 μg/day for children aged 1–3 years and 90 μg/day for those aged 4–6 years. In contrast, the control group consumed approximately 44 μg/day, based on repeated dietary analyses. Despite the elevated Se intake in the intervention group, clinical examinations revealed no signs of selenosis in either group [239] (Table 2).

## 10. Selenium as a Factor in Cognitive Well-Being: Results from Human Studies

According to human studies referenced in this research, low maternal selenium status during pregnancy was associated with reduced psychomotor performance in infants at six months of age and an increased incidence of infections during the first six weeks of life in a Norwegian population [240]. The study results demonstrated an inverted U-shaped relationship between Se levels in the first trimester of pregnancy and neurodevelopmental outcomes in both the mother and child at 12 months of age [241]. A study examining the relationship between neonatal brain size and Se levels in mother–child pairs revealed that perinatal selenium intake can influence brain length and width. The study also suggested that neonatal cerebellar size during pregnancy may serve as a potential biomarker of Se homeostasis [242]. A study evaluating the relationship between maternal Se levels and neurodevelopment at 5 years of age found an inverted U-shaped association: both low and high Se levels appeared to be detrimental to neurodevelopment. This suggests that the prenatal period may be particularly vulnerable to Se neurotoxicity [243]. Furthermore, prenatal selenium status has been reported to be associated with psychomotor abilities during the early years of a child’s life [244]. A study evaluating the influence of prenatal and childhood selenium status on child development in a cohort of 750 children found a positive association between maternal erythrocyte selenium (Ery-Se) levels during pregnancy and children’s language comprehension and psychomotor development at 1.5 years of age [245]. These findings suggest that adequate prenatal and childhood Se levels are crucial for long-term brain development [246]. A systematic review assessed the association between seafood consumption during pregnancy and neurocognitive development, utilizing methodologies outlined by the Dietary Guidelines for Americans 2020–2025 Science Advisory Committee. Understanding the impact of maternal seafood consumption on children’s neurocognitive outcomes has significant implications for public health [247].

## 11. Selenium Status in Pregnant Women: Implications for Child Health

Se plays a critical role in reproductive processes, particularly in women. Numerous studies have explored whether Se deficiency during pregnancy affects the developmental outcomes of children. Evidence from the literature indicates that Se deficiency is prevalent during pregnancy, and insufficient micronutrient intake during this critical period has been associated with impaired fetal growth, suboptimal development, and adverse pregnancy outcomes [248]. The findings of this study highlight the critical role of Se in supporting healthy fetal development during normal pregnancy. A clear association was observed between maternal Se status and dietary Se intake, as well as between serum Se levels, GPX activity, and various neonatal anthropometric parameters, including birth weight, length, and Apgar scores. Furthermore, similar correlations were identified among women with normal pregnancies [249]. Moreover, a systematic review and meta-analysis demonstrated an association between the micronutrient status of Se, zinc, and copper during pregnancy and birth weight. The findings highlighted significant correlations between selenium and zinc levels in maternal blood and umbilical cord blood with neonatal birth weight [250]. A recent study investigating iodine and selenium status in mothers and infants from a birth cohort in northern Sweden (n = 604) found a significant correlation between selenium levels in infant red blood cells and those in maternal red blood cells during pregnancy (ρ = 0.38, *p* < 0.001). These findings suggest that the transport of iodine and selenium to the fetus and infant is prioritized during gestation and early development [251]. A subsequent study revealed that women with the lowest selenium concentrations in maternal serum during the 10th–14th weeks of pregnancy (quartile Q1) had an approximately threefold higher risk of delivering a baby with a birth weight below the 10th percentile compared to women with higher selenium concentrations (quartiles Q2–Q4) [252]. A recent study examined the association between Se biomarkers of selenium status, birth weight, and infant growth outcomes (age-adjusted length, weight, head circumference, and weight-for-length z-score) at birth, 1 year, and 2 years of age among a cohort of pregnant and lactating women in Dhaka, Bangladesh. A small association between infant growth and venous cord (VC) and whole blood selenium (WBSe) (length-for-age z-score β, 95% CI, at birth: −0.05 (−0.1, −0.01); 12 months β: −0.05 (−0.08, −0.007)) was determined. Weight-for-age z-score was also associated with WBSe at birth (birth: −0.07 (−0.1, −0.02); 12 months: −0.05 (−0.1, −0.005)) and WBSe VC (birth: −0.05 (−0.08, −0.02); 12 months: −0.05 (−0.09, −0.004)). These results suggest the possibility that both in utero and postnatal WB and serum selenium may negatively influence infant growth outcomes [253]. Additionally, recent studies suggest that low maternal Se levels during pregnancy may be associated with decreased thyroid function and have also been reported to be associated with low birth weight [254]. A study involving 719 mother–child pairs suggests that Se deficiency in children, influenced by maternal Se status, may serve as a risk factor for the development of ADHD and ASD. The findings indicate that inadequate Se intake during pregnancy could contribute to an increased likelihood of ADHD and ASD in offspring [255]. Also, a recent report by Polanska et al. assessed the prevalence of Se deficiency among 683 children aged 6–59 months and women of reproductive age (n = 683) in sub-Saharan Africa, showing that mothers with Se-deficient children were 4 times more likely to have Se-deficient children than Se-adequate children and 3 times more likely to have Se deficiency in girl children, and that maternal selenium deficiency increased the risk of selenium deficiency in children [256]. Se plays a multifaceted role during pregnancy, with numerous studies suggesting a possible link between Se and early neurodevelopment.

A study demonstrated that low prenatal Se levels negatively impact children’s language development and psychomotor skills [140]. A similar cohort study identified a positive correlation between maternal plasma Se levels during pregnancy and language and motor development in Polish children in the first years of life [141]. Furthermore, the current study identifies a potential association between prenatal Se levels and the development of psychomotor skills during the early years of a child’s life [244]. Moreover, previous studies have identified an inverted U-shaped relationship between maternal Se concentrations and neuropsychological development in children, based on an analysis of 650 mother–child pairs [241]. A nonlinear association between maternal Se status and neuropsychological development in 5-year-old children has also been observed in a Spanish cohort [243]. The findings of Skröder et al. demonstrated that maintaining an adequate Se status during both fetal development and childhood positively influences cognitive function in children and revealed a significant positive association between prenatal Se levels and cognitive performance in children aged 5 and 10 years [246]. A study by Močenić et al. showed that perinatal Se intake can alter brain length and width in a study of the relationship between Se levels in neonatal brain sizes and mother–infant pairs [242]. The study findings indicated that, in a Norwegian population, low maternal Se levels were linked to reduced psychomotor performance at 6 months of age and a higher incidence of infant infections during the first 6 weeks of life [240]. This study investigated the associations of maternal Se intake from diet and supplements during the first half of pregnancy in a cohort of 71,728 women. An increase of one standard deviation in maternal dietary selenium intake was linked to higher birth weight z-scores (ß = 0.027, 95% CI: 0.007–0.041) and a reduced risk of small-for-gestational-age (SGA) births (OR = 0.91, 95% CI: 0.86–0.97). These findings suggest that a Se-rich maternal diet during pregnancy may support optimal fetal growth [257]. According to this study, the low and insufficient Se status of pregnant women in central Poland is directly reflected in the poor Se status of newborns [258].

## 12. Selenium Supplementation and Child Growth Outcomes

A meta-analysis of 15 randomized controlled trials, encompassing a total of 2931 patients, demonstrated that all five types of Se-based nutritional medical adjuncts (NMAs) yielded significantly greater improvements in metaphyseal radiographic outcomes compared to placebo. Among the examined interventions, selenium salts exhibited the highest efficacy, followed by sodium selenite combined with vitamin E, Se-enriched yeast, sodium selenite alone, and sodium selenite combined with vitamin C. These findings highlight the potential of Se supplementation in promoting the repair of metaphyseal lesions [259]. Similar studies, including 11 community-based meta-analyses involving 2652 participants from 5 provinces in China, revealed that high Se supplementation effectively reduces the incidence of new-onset diseases in healthy children. Moreover, it has been shown to be clinically effective in enhancing the recovery of metaphyseal and phalangeal lesions in children diagnosed with Keshan–Beck disease [260]. Also, based on this study, it was observed that an increase in selenium concentration in blood plasma during the acute phase of the systemic inflammatory response correlates with the duration of ventilation in children and a reduction in their length of stay in the intensive care unit, which can be the basis for trials on the effect of selenium supplementation [261]. This study identified a significant negative correlation between hair Se levels (r = −0.785) and the prevalence of Kashin–Beck disease (KBD) among Se-deficient children. The findings underscore that adequate Se supplementation could potentially mitigate the prevalence of KBD [262].

## 13. Future Directions

Se is a vital trace element essential for children’s growth, neurodevelopment, and thyroid and immune system function. It is also involved in the regulation of redox mechanisms, neuroinflammation, and controlled oxidative stress, all of which are integral to immune responses and various cellular processes through the accumulation of selenoproteins. Studies on the effects of Se deficiency and toxicity on the human body, as well as Se deficiency and maternal Se status, offer valuable insights into the timing and dosage required to support optimal growth, neurodevelopment, and immune functionality in children. Despite its established significance in numerous biological processes, the precise molecular mechanisms by which selenium influences gene expression, protein synthesis, and cellular function during critical stages of development remain inadequately understood. Identifying the molecular pathways through which cells interact with Se is essential to uncovering its specific roles in metabolic regulation during early development. Se’s contribution to neurodevelopment warrants special attention, as emerging evidence suggests its potential influence on brain growth, cognition, and behavior during childhood. Future research should aim to expand our understanding of the multifaceted role of Se in childhood development. This includes developing evidence-based strategies to optimize Se intake to help promote growth, strengthen immunity, and improve cognitive outcomes. Furthermore, identifying the benefits of Se supplementation could have profound public health implications, contributing to interventions aimed at preventing developmental deficits and supporting overall child well-being.

## 14. Conclusions

In conclusion, Se is a crucial trace element essential for child growth, neurodevelopment, and immune function, including bone metabolism. Selenoproteins regulate oxidative stress, thyroid hormone metabolism, and immune responses. Studies indicate that adequate Se levels support physical growth, cognitive function, and immunity, while deficiency is linked to growth retardation, neurodevelopmental delays, and immunosuppression. Optimizing Se intake through diet or supplementation can enhance child health outcomes. Future research should clarify its mechanisms, define optimal intake levels, and explore interactions with other micronutrients. Public health efforts targeting Se deficiency, especially in vulnerable populations, are crucial for ensuring optimal child development.

## Figures and Tables

**Table 1:** The role of Selenium (Se) supplementation in animal models.

Duration of Selenium Treatment	Se Type	Se Dose	Model	Main Target	Effects of Selenium	References
2 weeks 6 weeks	Se-enriched yeast (SeYeast) Sodium Selenite (Na_2_SeO_3_)	0.15 mg/kg 0.01 ppm	Chicken Mice	Tonsils and spleen Immune system	Induces higher expression of antiviral immunity genes Regulates immune system responses via IFN-γ and IL-6	[224,225]
38 days	Organic Se-enriched yeast (Se yeast)	0.32 mg/kg	Pig	Immune system	Organic Se positively influenced the pigs’ immune response and antioxidant capacity	[226]
42 days	Organic selenium	0.30 ppm	Chicken	Growth Performance	Improved growth	[227]
4 weeks	SeMet	0.6 mg/kg	Chicken	Muscle	Improved growth performance	[228]
18 days	Selenium yeast (SY)	0.45 mg/kg	Chicken	Eggshells	Enhanced eggshell strength	[229]
14 days	Selenium yeast (SY)	0.133 mg/kg	Chicken	Growth performance	Enhanced growth efficiency	[230]

**Table 2:** Effects of selenium (Se) deficiency in children.

No. of Participants	Age of Participants	Study Design	Effects of Deficiency	Outcome	References
541	6–60 months	Randomized controlled trial	Adverse effect on children’s cognitive abilities	Impairment of cognitive skills	[179]
628	54–64 months	Cross-sectional	Negatively affects thyroid hormone regulation	Impaired antioxidant defense of the thyroid gland	[217]
14823	7–12 years	Cross-sectional	Disrupts the regulation of immune responses	Changes in gut microbiota	[218]
150	6–18 years	Observational	Disrupts the normal development and functioning of the body	Kashin–Beck disease	[219]
8	7–12 years	Cross-sectional	Pathological alterations in articular cartilage	Kashin–Beck disease	[220]
